# Editorial: The importance of Th17/Treg imbalance in asthma and COPD development and progression

**DOI:** 10.3389/fimmu.2022.1025215

**Published:** 2022-09-23

**Authors:** Fernanda Degobbi T. Q. S. Lopes, Iolanda de Fátima Lopes Calvo Tibério, Adriana Leme, Lucy Fairclough

**Affiliations:** ^1^ Department of Medicine, University of São Paulo, São Paulo, Brazil; ^2^ School of Medicine, University of Pittsburgh, Pittsburgh, PA, United States; ^3^ Department of Immunology, University of Nottingham, Nottingham, United Kingdom

**Keywords:** copd, asthma, TH17/Treg response, inflammatory control, pro-inflammatory microenvironment

In the current Research Topic of *Frontiers in Immunology*, clinical and experimental studies describe the Th17/T regulatory cell imbalance in both COPD and asthma, highlighting how a failure in inflammatory control mediated by T regulatory (Treg) cells can affect the physiopathology of these lung diseases.

CD4+ T cells, depending on the cytokines present in the microenvironment, can differentiate into T-helper subtypes (Th1, Th2, Th17, and T-regulatory cells) (1). Most studies in asthma describe a central role of Th2 cells and their related cytokines (IL-4, IL13, and IL-5) (León et al.). In COPD, Th17 cells and IL-17 have been described both in the disease progression as well as in bacterial infections that lead to COPD exacerbation (2). Only in recent years have Th17 cells been also described in airways of patients with asthma who also showed increased amounts of neutrophils and did not respond to classical therapy (3, 4).

Treg cells are recognized by suppressing inflammatory responses through anti-inflammatory cytokine release such as IL-10 and transforming growth factor-B (TGF-b) (1). Additionally, some studies have demonstrated the importance of these cells as well as their anti-inflammatory interleukins in preventing the development and progression of asthma and COPD (5, 6, Dai et al.; Shen et al.).

Clinical and experimental studies in COPD do not show a decrease in Treg cell levels, but lower expression of IL-10 is often observed (1). In a temporal study, Ito et al. (7) detected a decrease in IL-10 positive cells in parenchyma followed by an increase in IL-17 positive cells. Additionally, they demonstrated a decrease in Treg cells only in the first stages of COPD development. Latterly, the level of these cells reaches the values observed in control groups, with no COPD induction. These data corroborate with a study from Cervilha DAB et al. (2), which showed in an experimental model of COPD exacerbation that the inflammatory response was characterized by an increase in Th17 cytokine levels and a decrease in IL10 positive cells, even though there was Treg cell differentiation. Furthermore, Lourenço JD et al. (1) evaluated the gene expression of intracellular proteins and related cytokines involved in Th17/Treg responses in both mild and moderate COPD patients, comparing local and systemic responses. They showed that intracellular signaling for the Th17 response was present at the early stages of this disease. Th17 markers could be found in lung samples from mild COPD, whereas, in moderate stages, they were observed also in blood samples. Regarding the Treg response, they showed that despite the increase in markers for Treg cell differentiation in the different COPD stages in both lung and blood samples, a decrease in IL10 levels was detected in patients in advanced stages of this disease.

In an experimental model of asthma, de Brito et al. showed that a treatment with photobiomodulation increased the IL-10 release as well as Treg cells in the bronchoalveolar lavage, attenuating the inflammatory response's functional and structural changes. Previously, Camargo et al. (8) showed in a murine model exacerbated by LPS that the inhibition of IL-17 controlled the airway inflammation, remodeling, and the oxidative stress mechanism by the attenuation of the forkhead helix transcription factor (Foxp3) expression that has been considered a specific molecular marker for Treg cells.

The decrease in IL-10 expression that benefits the inflammatory development and progression is described in articles both in COPD and asthma, revealing that the total amount of Treg cells is not the key, but the functionality of these cells is central to any effect. Treg cell differentiation and development as well as the maintenance of their function depend on the Foxp3 expression. However, this transcription factor expression is also significantly upregulated in effector T cells, which do not have immunomodulatory capacity. Thus, the role of Foxp3 as a specific marker for Treg has been increasingly questioned, and it is still uncertain if there are specific molecular markers that are expressed only in Treg cells (9). Meanwhile, there are different clinical and experimental studies that are using a combination of different molecular markers expressed for Treg cells to identify their immunosuppressive activity.

Shen et al. showed that the pathogenesis of allergic asthma may be associated with CCR6+Treg cell recruitment. CCR6 is a G protein–coupled chemokine receptor expressed on several types of immune cells, including Th17 and Treg cells (10–12). These authors showed that there is an increase in CCR6+Treg cell migration from peripheral blood to the airways, probably *via* CCL20 signaling. However, in the airways, CCR6+Treg cells are incapable of attenuating allergic inflammation, since there is an increase in pro-inflammatory cytokines that leads to the conversion of Treg cells into Th17-like cells. Similarly, Dai et al. showed that a combination of ICS/LABA and subcutaneous immunotherapy reduced the percentage of Th17 cells and the serum IL-17 level, as well as decreased the Th17/Treg ratio in house dust mites (HDM) asthmatic allergic children. Furthermore, they observed elevated percentages of Treg cells and IL-10 levels in these patients.

Beyond the importance in defining the different immune cell subtypes, there is an increasing interest to better understand how molecular markers could define distinct stages of diseases, such as asthma. Considering that the importance of Th1, Th2, and Th17 in severe asthma has been described, Adel-Patient et al. performed the analysis of immune markers for these responses in blood and bronchoalveolar lavage fluid (BAL) comparing severe asthmatics with control subjects with chronic respiratory disorders other than asthma. After performing multivariate analysis on these data, they identified possible targets to characterize an immune signature for severe asthmatics. They found that children with severe asthma showed a mixed Th1/Th2 profile in BAL with non-neutrophilic environment that was clearly different from control subjects.

The recent findings described in this Research Topic reinforce the evidence that a failure in IL-10 release depends not only on Treg differentiation but mainly also on the cytokines present in the microenvironment that drives cell differentiation to determine the functionality of these cells. Further studies are necessary to better define the role of these different phenotypes in COPD and asthma development.

## Author contributions

FL wrote the original draft; IT, AL, and LF revised the final manuscript. All authors contributed to the article and approved the submitted version.

## Conflict of interest

The authors declare that the research was conducted in the absence of any commercial or financial relationships that could be construed as a potential conflict of interest.

## Publisher’s note

All claims expressed in this article are solely those of the authors and do not necessarily represent those of their affiliated organizations, or those of the publisher, the editors and the reviewers. Any product that may be evaluated in this article, or claim that may be made by its manufacturer, is not guaranteed or endorsed by the publisher.

## References

[1] Lourenço JD Teodoro WR Barbeiro DF Velosa APP Silva LEF AR M. Th17/Treg-related intracellular signaling in patients with chronic obstructive pulmonary disease: Comparison between local and systemic responses. Cells (2021) 10(7):1569. doi: 10.3390/cells10071569 34206428 PMC8305827

[2] Cervilha DAB Ito JT Lourenço JD Olivo CR Saraiva-Romanholo BM Volpini RA. The Th17/Treg cytokine imbalance in chronic obstructive pulmonary disease exacerbation in an animal model of cigarette smoke exposure and lipopolysaccharide challenge association. Sci Rep (2019) 9(1):1921. doi: 10.1038/s41598-019-38600-z 30760822 PMC6374436

[3] De Boever EH Ashman C Cahn AP Locantore NW Overend P Pouliquen IJ. Efficacy and safety of an anti-IL-13 mAb in patients with severe asthma: a randomized trial. J Allergy Clin Immunol (2014) 133(4):989–96. doi: 10.1016/j.jaci.2014.01.002 24582316

[4] Guan Y Ma Y Tang Y Liu X Zhao Y An L. MiRNA-221-5p suppressed the Th17/Treg ratio in asthma *via* RORγt/Foxp3 by targeting SOCS1. Allergy Asthma Clin Immunol (2021) 17(1):123. doi: 10.1186/s13223-021-00620-8 34863307 PMC8643019

[5] Sales DS Ito JT Zanchetta IA Annoni R Aun MV Ferraz LFS. Regulatory T-cell distribution within lung compartments in COPD. COPD (2017) 14(5):533–42. doi: 10.1080/15412555.2017.1346069 28745532

[6] Silva LEF Lourenço JD Silva KR Santana FPR Kohler JB Moreira AR. Th17/Tregimbalance in COPD development: suppressors of cytokine signaling and signal transducers and activators of transcription proteins. Sci Rep (2020) 10(1):15287. doi: 10.1038/s41598-020-72305-y 32943702 PMC7499180

[7] Ito JT Cervilha DAB Lourenço JD Gonçalves NG Volpini RA Caldini EG. Th17/Treg imbalance in COPD progression: A temporal analysis using a CS-induced model. PloS One (2019) 14(1):e0209351. doi: 10.1371/journal.pone.0209351 30629626 PMC6328193

[8] Camargo LDN Dos Santos TM de Andrade FCP Fukuzaki S Dos Santos Lopes FDTQ de Arruda Martins M. Bronchial vascular remodeling is attenuated by anti-IL-17 in asthmatic responses exacerbated by LPS. Front Pharmacol (2020) 11:1269. doi: 10.3389/fphar.2020.01269 33013361 PMC7500412

[9] Hou J Sun Y Hao Y Zhuo J Liu X Bai P. Imbalance between subpopulations of regulatory T cells in COPD. Thorax (2013) 68:1131–9. doi: 10.1136/thoraxjnl-2012-201956 23749814

[10] Shanmugasundaram U Bucsan AN Ganatra SR Ibegbu C Quezada M Blair RV. Pulmonary mycobacterium tuberculosis control associates with CXCR3- and CCR6-expressing antigen-specific Th1 and Th17 cell recruitment. JCI Insight (2020) 5(14):e137858. doi: 10.1172/jci.insight.137858 PMC7453885 32554933

[11] Kulkarni N Meitei HT Sonar SA Sharma PK Mujeeb VR Srivastava S. CCR6 signaling inhibits suppressor function of induced-treg during gut inflammation. J Autoimmun (2018) 88:121–30. doi: 10.1016/j.jaut.2017.10.013 29126851

[12] Cosmi L Maggi L Santarlasci V Capone M Cardilicchia E Frosali F. Identification of a novel subset of human circulating memory CD4(+) T cells that produce both IL-17A and IL-4. J Allergy Clin Immunol (2010) 125(1):222–30.e1-4. doi: 10.1016/j.jaci.2009.10.012 20109749

